# The value of resting-state functional MRI in subacute ischemic stroke: comparison with dynamic susceptibility contrast-enhanced perfusion MRI

**DOI:** 10.1038/srep41586

**Published:** 2017-01-31

**Authors:** Ling Ni, Jingwei Li, Weiping Li, Fei Zhou, Fangfang Wang, Christopher G. Schwarz, Renyuan Liu, Hui Zhao, Wenbo Wu, Xin Zhang, Ming Li, Haiping Yu, Bin Zhu, Arno Villringer, Yufeng Zang, Bing Zhang, Yating Lv, Yun Xu

**Affiliations:** 1 Department of Radiology, The Affiliated Drum Tower Hospital of Nanjing University Medical School, University of Nanjing, Nanjing, China; 2 Department of Neurology, The Affiliated Drum Tower Hospital of Nanjing University Medical School, Nanjing, China; 3 Department of Radiology, The Affiliated Drum Tower Hospital of Nanjing Medical University, Nanjing, China; 4 Department of Radiology, Mayo Clinic and Foundation, Rochester, MN, USA; 5 Max Planck Institute for Human Cognitive and Brain Sciences, Leipzig, Germany; 6 Center for Cognition and Brain Disorders, Affiliated Hospital, Hangzhou Normal University, Hangzhou, Zhejiang, China; 7 Zhejiang Key Laboratory for Research in Assessment of Cognitive Impairments, Hangzhou, Zhejiang, China

## Abstract

To evaluate the potential clinical value of the time-shift analysis (TSA) approach for resting-state fMRI (rs-fMRI) blood oxygenation level-dependent (BOLD) data in detecting hypoperfusion of subacute stroke patients through comparison with dynamic susceptibility contrast perfusion weighted imaging (DSC-PWI). Forty patients with subacute stroke (3–14 days after neurological symptom onset) underwent MRI examination. Cohort A: 31 patients had MRA, DSC-PWI and BOLD data. Cohort B: 9 patients had BOLD and MRA data. The time delay between the BOLD time course in each voxel and the mean signal of global and contralateral hemisphere was calculated using TSA. Time to peak (TTP) was employed to detect hypoperfusion. Among cohort A, 14 patients who had intracranial large-vessel occlusion/stenosis with sparse collaterals showed hypoperfusion by both of the two approaches, one with abundant collaterals showed neither TTP nor TSA time delay. The remaining 16 patients without obvious MRA lesions showed neither TTP nor TSA time delay. Among cohort B, eight patients showed time delay areas. The TSA approach was a promising alternative to DSC-PWI for detecting hypoperfusion in subacute stroke patients who had obvious MRA lesions with sparse collaterals, those with abundant collaterals would keep intact local perfusion.

Perfusion imaging has served an important role in directing therapeutic intervention in acute stroke patients, either during patient selection, such as identifying likely treatment-responders according to the existence of ischemic penumbra, or in adjusting the therapy based on patient response^1^,^2^,^3^,^4^. The penumbra tissue is assumed to survive up to about 36 hours^5^ and become part of the infarcted tissue if not salvaged. Thus, the long-lasting hypoperfusion during the subacute phase was supposed not to be penumbra but the overestimated benign oligemia, which would be unlikely to become part of the infarction^6^. However, perfusion deficits can lead to abnormal brain function^7^. Hillis and colleagues considered that the hypoperfused cortical regions that persisted for a few days after stroke onset were responsible for language or cognitive deficit, even when (diffusion weighted imaging) DWI showed no infarct or only small subcortical infarct, and thus urgent treatment such as carotid endarterectomy (CEA) was advocated rather than waiting for the traditional treatment opportunity^8^.

Moreover, hypoperfusion volume was considered as a marker of response to treatment aimed to improve cerebral perfusion in the subacute phase of ischemic stroke. One study^9^ found an association between improved perfusion of peri-infarct regions and restored function after blood pressure elevation therapy in patients with perfusion/diffusion mismatch up to seven days or more after the onset of symptoms^10^. They also suggested that reperfusion may be effective in the subacute phase of ischemic stroke if there is still a substantial area of salvageable tissue identified by diffusion/perfusion mismatch. Thus, with the help of perfusion imaging, the window of opportunity for intervention could be extended from the currently accepted 3–6 hours^11^ to several days, and the effect of the ongoing intervention could be monitored.

Various MR imaging techniques have been developed to assess cerebral hemodynamics. Dynamic susceptibility contrast perfusion weighted imaging (DSC-PWI) is the most commonly used in clinical practice. However, DSC-PWI requires administration of a contrast agent, which has been dose-restricted because of its association with nephrogenic systemic fibrosis and neuronal tissue deposition even in those with normal renal function^12^,^13^. In addition, the use of a contrast agent prohibits the acquisition of repeated scans during the same session, which can be necessary in a clinical setting because of data loss (e.g. from excessive motion), and sessions in different stages after stroke onset for monitoring functions. The potential side effects of contrast agents demand noninvasive alternatives. Arterial spin labeling (ASL) takes advantage of magnetically labeled blood water as an endogenous tracer for noninvasive quantification of brain perfusion. However, ASL in ischemic stroke may potentially underestimate cerebral blood flow (CBF)^14^ and have poor signal to noise ratios in situations of long transit times^15^, which limit its clinical application in identifying the perfusion deficits after stroke onset. Resting-state functional magnetic resonance imaging (rs-fMRI) using Blood oxygenation level-dependent imaging (BOLD) is a noninvasive imaging technique which does not require contrast agent application and maintains high temporal resolution. Moreover, BOLD is sensitive to local background hemodynamics changes^16^, and thus is a potential option to monitor perfusion changes after stroke onset. Recently, time-shift-analysis (TSA) based on BOLD data has been applied on acute ischemic stroke^17^, and on chronic hypoperfusion patients^18^ and the time delay areas in each were proven to be comparable to DSC-PWI.

Given that BOLD technique is available for almost every scanner of 1.5 T or 3 T, TSA holds great potential for clinical practice. Therefore, its value of estimating hypoperfusion should be repeated and validated. In our present study, we applied TSA combined with the DSC-PWI and MRA methods on subacute stroke patients, part of which having both BOLD scanning and DSC-PWI and the other part having only BOLD data, to further validate that rs-fMRI BOLD was able to detect perfusion injury in subacute stroke patients, thus had important implications for monitoring the perfusion status and establishing therapeutic regimen.

## Results

Hypoperfusion lesions were detected in scans of twenty-two patients. The characteristics of the subacute stroke patients are summarized in Tables 1 and 2. The average time to peak (TTP) difference between the ipsilateral hypoperfusion areas and corresponding contralateral healthy regions ranged from 0.10 s to 4.43 s (2.30 ± 1.32 s). Mean time course from whole brain and healthy hemisphere (with normal DWI appearance) contralateral to the lesion, respectively, were calculated as reference time course. The time delay areas based on these two references were sizable and the volumetric results were listed in Tables 1 and 2.

For those 31 patients with combined DSC-PWI and BOLD data (cohort A), 15 patients had intracranial large-vessel occlusion or stenosis confirmed by MRA. Fourteen patients with sparse collaterals showed time delay areas from TSA, which were comparable to those on their TTP maps from DSC-PWI, one with abundant collaterals showed neither TTP nor TSA time delay. The remaining 16 patients without obvious MRA lesions showed neither TTP nor TSA time delay. The perfusion lesion sizes from TSA were larger (not all) than those obtained by DSC-PWI, the group average spatial overlap, i.e., Dice coefficient (DC), of the two measurements was DC_(H)_ 0.58 ± 0.13 (range, 0.35–0.78) and DC_(G)_ 0.59 ± 0.12 (range, 0.36–0.77), respectively (Table 1).

**Figure 1:**
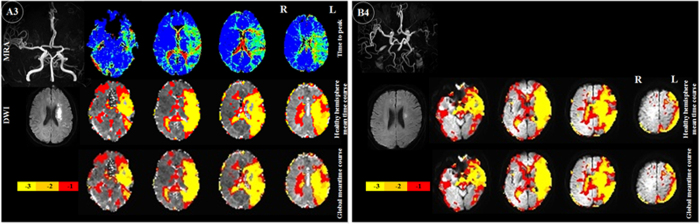
Perfusion injury in subacute stroke patients with left middle cerebral artery (MCA) severe stenosis/occlusion. A3, Comparison of time delay maps from global mean time course (bottom row), healthy hemisphere mean time course (middle row) and time to peak (TTP, upper row). TTP and TSA demonstrated comparable hypoperfusion areas. A3&B4, the time delay areas acquired based on global and healthy hemisphere mean time course were sizable. MRA = magnetic resonance angiography, DWI = diffusion weighted imaging. −1, −2, −3 in color bar indicate −1TR, −2TR, −3 TR time shift.

**Figure 2:**
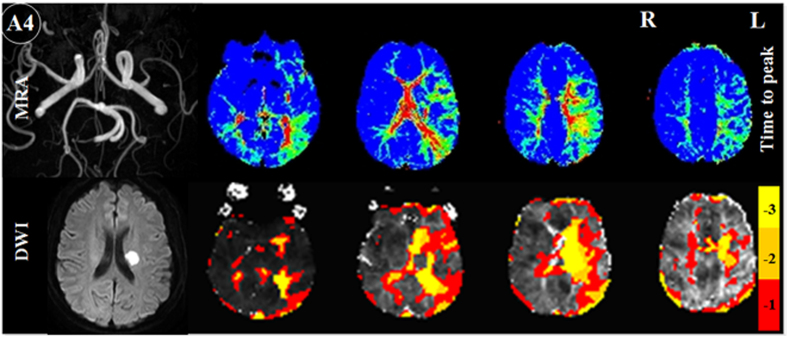
Perfusion injury detected by time to peak (TTP) on dynamic susceptibility-weighted contrast-enhanced perfusion images (top right) and TSA on resting-state functional MR images (bottom right) in subacute stroke patient with obvious thinness along the whole course compared to the right MCA. TTP delay was found in the left MCA corresponding blood supply area. TSA also demonstrated pronounced comparable time delay areas. MRA = magnetic resonance angiography, DWI = diffusion weighted imaging. −1, −2, −3 in color bar indicate −1TR, −2TR, −3 TR time shift.

**Figure 3:**
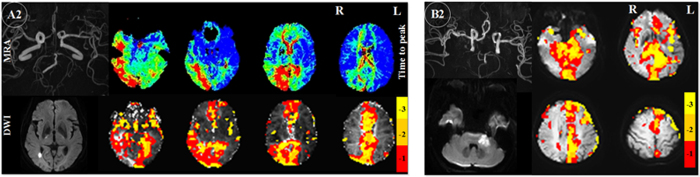
Perfusion injury in subacute stroke patients with severe stenosis/occlusion of the anterior cerebral artery (ACA), basilar artery (BA) and posterior cerebral artery (PCA). Hypoperfusion areas from time shift analysis (TSA) and time to peak (TTP) (A2), TSA (B2) maps were found in the corresponding blood supply areas of these arteries, although infarctions were only found in the right corona radiata, basal ganglia and occipital lobe (A2) and left brachium pontis (B2). MRA = magnetic resonance angiography, DWI = diffusion weighted imaging. −1, −2, −3 in color bar indicate −1TR, −2TR, −3 TR time shift.

**Figure 4:**
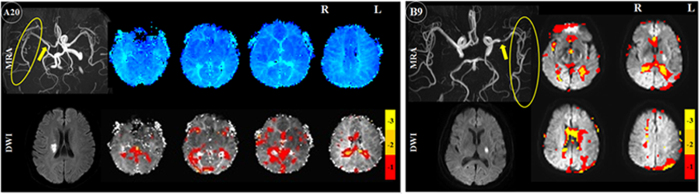
Patient A20 and B9, who had severe MRA stenosis/occlusion (yellow arrows) with abundant collaterals (yellow circles), no time delay regions were detected in the corresponding blood supply territory on TSA (A20, B9), nor TTP delay on DSC-PWI (A20). MRA = magnetic resonance angiography, DWI = diffusion weighted imaging. −1, −2, −3 in color bar indicate −1TR, −2TR, −3 TR time shift.

**Figure 5:**
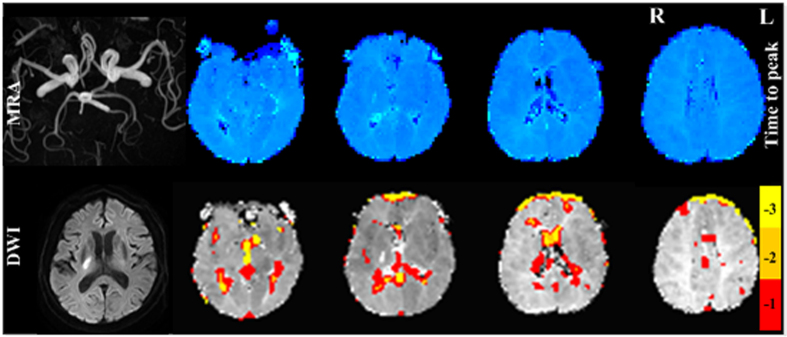
A single example patient among those who had no obvious MRA lesions, there were neither TTP delay regions nor time delay areas from TSA. MRA = magnetic resonance angiography, DWI = diffusion weighted imaging. −1, −2, −3 in color bar indicate −1TR, −2TR, −3 TR time shift.

Seven patients had left middle cerebral artery (MCA) and two had right MCA lesions on MRA: there was severe stenosis/occlusion in patient A3, 6, 7, 9, 11, 13 and 14 (left), A8, 12 (right), and obvious thinness along the whole course compared to the right MCA in patient A4. TTP delay was found in the left MCA’s corresponding blood supply area, and TSA also demonstrated pronounced comparable time delay areas (Fig. 1. A3 and Fig 2). MRA in patient A2 and 10 showed severe stenosis/occlusion in basilar artery (BA) and/or the right anterior cerebral artery (ACA), bilateral posterior cerebral artery (PCA). Hypoperfusion areas from the TTP map were found in the corresponding blood supply areas of these arteries, although infarctions were only found in the right corona radiata, basal ganglia and occipital lobe. The areas that showed time delays relative to the reference time course corresponded to the areas of hypoperfusion as identified by TTP maps (Fig. 3.A2). Patient A1, 5 and 20 suffered occlusion in the intracranial segment of the left internal carotid artery (IS-ICA). A1 and 5 with sparse collaterals showed time delay areas from TSA, which were comparable to those on their TTP maps from DSC-PWI. A20, who had abundant collaterals showed neither TTP nor TSA time delay (Fig. 4. A20). In the other 16 patients who had no obvious lesions on MRA, there were neither TTP delay regions nor time delay areas from TSA (Fig. 5).

For cohort B of nine patients who had intracranial large-vessel occlusion or stenosis with BOLD data only, eight patients showed time delay areas from TSA, and one patient who suffered from lacunar infarction in the left basal ganglia with severe stenosis of the left MCA did not show time delay in the corresponding area.

In patient B1, 3, 4, 6, 7 and 8 who had similar arteriostenoses to patient A3, 6, 7, 9, 11, 13 and 14: severe stenosis of left MCA, TSA demonstrated pronounced time delay areas in the left MCA territory (Fig. 1.B4). In addition, patient B1, who also had mild stenosis in the right MCA, did not show time delay areas from TSA in the right hemisphere. In patient B5, who suffered severe stenosis of the BA, the areas which showed time delays compared to the reference time course corresponded to the blood supply territory. Like patient A2, MRA showed severe stenosis/occlusion in left A1, BA and right P1 in patient B2. Hypoperfusion areas from the TSA map were found in the corresponding blood supply territory, although infarctions were only found in left brachium pontis (Fig. 3.B2). Patient B9, who had severe stenosis of the left MCA with abundant collaterals, did not show time delay region in the corresponding blood supply territory (Fig. 4.B9).

## Discussion

In this study, we aimed to use the rs-fMRI TSA approach to identify hypoperfusion lesion in patients with subacute stroke. We successfully detected hypoperfusion areas by the TSA approach in patients with intracranial large-vessel occlusion or severe stenosis with sparse collaterals, which were comparable to the TTP delay areas from DSC-PWI. The perfusion lesion sizes from TSA were larger than those obtained by DSC-PWI, the group average spatial overlap was DC_(H)_ 0.58 ± 0.13 (range, 0.35–0.78) and DC_(G)_ 0.59 ± 0.12 (range, 0.36–0.77), respectively. In those who had unremarkable or mild stenosis, no perfusion lesions were identified in neither the TTP map nor the TSA delay methods. Overall, 100% comparable positive and negative results between TSA and TTP based on DSC-PWI could be concluded in the present study. Moreover, when we retested the detection rate of the TSA method in a new cohort of subacute stroke patients who suffered from severe stenosis/occlusion of intracranial large-vessels, the high detection rate (88.9%) of hypoperfusion areas by using TSA in the corresponding blood supply territory was further identified.

BOLD signals usually reflect spontaneous neuronal activity during rs-fMRI or special brain activation induced by a task^19^. Moreover, the BOLD signals additionally contain information concerning local blood flow and oxygen consumption^20^, and thus impaired hemodynamic status or severity of hemodynamic impairment could be assessed by evaluating the changes in BOLD response, which can be absent, reduced, negative or delayed (time shift/lagged)^21^,^22^. TSA was defined as the time shift necessary for maximum correlation with an average representative time series (i.e. the global or contralateral healthy brain mean). Such an approach has recently been used to assess pathophysiological events associated with hemodynamics and provided a high spatial correspondence on the individual level with the area of hypoperfusion as defined by DSC-PWI without the application of contrast agent^17^,^18^. TTP has been used to determine the extent of hypoperfused brain in large case series and clinical trials^23^. In our study, the average TTP delay between the ipsilateral hypoperfusion area and corresponding contralateral healthy regions ranged from 0.10 s to 4.43 s, suggesting that the time shift range from -3TR to + 3TR ( ± 6.0 s) which we employed for TSA was reasonable.

One major finding in our present study was that for patients with combined DSC-PWI and BOLD data, patients with intracranial large-vessel occlusion or stenosis with sparse collaterals confirmed by MRA showed considerable time delay areas, while one with abundant collaterals and the remaining patients without MRA lesions/vessel occlusion or stenosis showed no delay versus the reference time course, indicating that the time delay i.e. hypoperfusion was more likely to be detected in stroke patients with intracranial large-vessel occlusion or stenosis with sparse collaterals^7^. This was partially consistent with one study^24^ focused on acute stroke, which also concluded that PWI/DWI mismatch was always associated with a major vessel occlusion. For patient B1, who had both severe stenosis in the left MCA and mild stenosis in the right MCA, we only detected time delay areas in the left MCA blood supply territory, which might further confirm that the hypoperfusion was more likely to appear in stroke patients with intracranial large-vessel occlusion or stenosis, rather than in those with mild stenosis.

The distribution of hypoperfusion was more dependent on MRA lesions rather than DWI infarction^7^. Like patient A2 and B2, who suffered severe stenosis in multiple arteries, time delay areas and TTP delay areas were found in the corresponding blood supply territory, but not simply surrounding the infarctions from the DWI map. Moreover, all of the time delay areas detected by TSA were similar to those perfusion injuries identified by the TTP map from DSC-PWI, and the delineation of abnormal areas appeared more conspicuous on the TSA map than on the TTP map, which might suggest that the TSA approach could clearly detect the boundary of hypoperfusion. As the hypoperfusion ROIs were traced manually by two radiologists, there might be some errors in manual measurement, resulted in the widely varying DC values, improved quantitative measurements were urgently required in the future study. The perfusion lesion sizes from TSA in our study were larger (not all) than those obtained by DSC-PWI, which might be attributed to that TSA was susceptible to longer paths of blood flow due to collaterals but still intact local perfusion^7^.

The second important finding in this study was that in order to retest the detection rate of hypoperfusion by TSA, we enrolled a new cohort with 9 subacute stroke patients who had obvious MRA lesions with only BOLD but no DSC-PWI data, in which time delay areas were detected in 88.9% patients in the corresponding blood supply territory using the TSA method. Only one patient, who suffered from lacunar infarction in the left basal ganglia with severe stenosis of left middle cerebral artery, did not show time delay in the corresponding area. The exact interpretation for this patient was uncertain, and might be due to the small size of the lesion. However, perfusion abnormalities have been observed in lacunar infarction during acute^25^ and subacute stroke^26^. This might also be ascribed to the abundant collaterals, similar to A20 (who suffered occlusion in the IS-ICA with abundant collaterals showed no hypoperfusion areas neither on TTP nor TSA maps), as the distal branches of this left MCA were well-visualized (Fig. 4. B9). Although no DSC-PWI was acquired for confirmation in these nine patients, we tentatively speculated that the TSA method had the potential to predict the perfusion abnormality of stroke patients who had intracranial large-vessel occlusion or stenosis with sparse collaterals during the subacute stage.

Our study had several limitations. First, although the stroke patients during the subacute phase were relatively stationary during the scan when compared with those during the acute phase, some patients still needed to be excluded from further analysis due to head motion artifacts, because the BOLD signal was severely affected. In addition, the 8-minute scanning time of rs-fMRI in our study was longer than conventional DSC-PWI, which only needed 2 minutes, and this long scanning time would increase the probability of head motion. Lv^17^ and Amemiya *et al*.^18^ reported that the acquisition time could be reduced to ~5 min, and still showed results similar to those of the full scan session. Thus, to be clinically viable, effective ways to manage head motion, such as prospective motion correction during scanning and reducing scanning time of data acquisition, would be urgently required in future study. Another limiting factor in this study was that the sample size was relatively small and the span of time after symptom onset was relatively long (3–14 days), and so the reliability and stability of the TSA approach applied on stroke requires further confirmation with more subjects at different stages of stroke.

In conclusion, 100% comparable positive and negative results between TSA and TTP based on DSC-PWI could be concluded in the present study. The TSA approach was a promising alternative to DSC-PWI for detecting perfusion injury in subacute stroke patients who had large-vessel occlusion or stenosis with sparse collaterals, those with abundant collaterals would keep intact local perfusion.

## Material and Methods

### Subjects

This prospective study was approved by the ethical committee of the Affiliated Drum Tower Hospital of Nanjing University Medical School, and all experiments were performed in accordance with relevant guidelines and regulations. After a detailed explanation of the study procedures, all subjects or their guardians signed an informed consent form before the study. From January 2014 to July 2016, forty-six stroke patients were consecutively enrolled in this study. All of them were recruited from patients hospitalized at the Affiliated Drum Tower Hospital of Nanjing University Medical School, Nanjing, China. They showed a persistent restricted area/high signal on DWI up to fourteen days after the onset of symptoms. The following exclusion criteria were applied in this study: (a) a contraindication to MRI, (b) any hemorrhagic infarction including subarachnoid hemorrhage, (c) concurrent chronic infarction, (d) head motion larger than 3.0 mm or 3.0° during MR scanning, (e) time to MRI scan less than 3 days after symptom onset. On the basis of these criteria, 6 patients were excluded owing to head motion more than 3.0 mm or 3.0° (n = 4), Moyamoya disease (n = 1), and subarachnoid hemorrhage (n = 1), and the data sets from the remaining 40 patients in the subacute phase of stoke were included in the final analysis. Among them, 31 patients underwent both DSC-PWI and BOLD data acquisition were defined as cohort A, and the remaining 9 patients had only BOLD data acquisition were defined as cohort B. All of these patients were treated with only conventional management such as antiplatelet, lipid-lowering, scavenging oxygen free radicals and neuroprotective agents but no intravenous thrombolysis or endovascular therapy before MRI acquisition.

### MRI data acquisition

MRI was performed within a range of 3 to 14 days after onset of neurological symptoms. MRI data was acquired on a 3 Tesla MR scanner (Achieva 3.0 T TX dual Medical Systems; Philips Medical Systems, Eindhoven, Netherlands). All subjects were placed in an eight-channel phased array head coil and fitted to foam padding to reduce head motion, and a pair of earplugs was used to reduce scanner noise. They were instructed to hold still and keep eyes closed but be awake during MRI acquisition. Compliance to these instructions was verified as part of the exit interview. The MR scanning protocol included diffusion-weighted imaging, BOLD, DSC-PWI, and conventional MRI (T1-weighted images, T2-weighted images, fluid attenuated inversion recovery (FLAIR) images, and 3D time-of-flight MR angiography). DWI was performed using a multislice, isotropic, single-shot EPI sequence, with B_max_ of 1,000 sec/mm^2^, repetition time msec/echo time msec, 2200/56; matrix, 192 × 109; field of view, 230 × 220 mm^2^; flip angle, 90°; slice thickness, 6.0 mm. A gradient-echo echo-planar (GRE-EPI) sequence sensitive to BOLD contrast was used to acquire functional images (repetition time msec/echo time msec, 2000/30; matrix, 64 × 64; field of view, 192 × 192 mm^2^; flip angle, 90°; slice thickness, 4.0 mm). Each brain volume comprised 35 axial slices and each functional image contained 240 volumes. Each fMRI scan lasted 480 s. DSC-PWI was acquired using a gradient-echo echo planar imaging sequence: repetition time msec/echo time msec, 2000/30; matrix, 96 × 95; field of view, 224 × 224 mm^2^; flip angle, 90°; slice thickness, 4.0 mm; duration = 88 s).

### Data analyses

#### Data preprocessing

Functional MR imaging data were preprocessed and analyzed using Data Processing Assistant for Resting-State fMRI Advanced edition (DPARSFA_V2.2; ref. 27; www.restfmri.net) on the MATLAB (The MathWorks, Inc., Natick, MA, USA) platform. The first ten volumes were discarded to allow for signal equilibrium and participants’ adaptation to the scanning circumstance. Slice timing correction and realignment were conducted for interleaved acquisition and head motion correction, respectively. Four patients were excluded because of excessive head motion (more than 3.0 mm maximum translation in any of the x, y or z directions, or 3.0 degree of maximum rotation about three axes) during scanning. The remaining data were spatially smoothed by convolution with an isotropic Gaussian kernel of FWHM 6 mm, removed the linear trend, and then band pass filtered (0.01–0.1 Hz). In order to overlay BOLD data results in images with lesion areas, the DWI and the TTP map from DSC-PWI data were coregistered to the individual’s mean functional image.

#### Time-shift-analysis

The effect of head motion (three rigid body translations and three rotations) from the BOLD data was regressed out, and the mean signal of global and healthy hemisphere (with normal DWI appearance) contralateral to the lesion, respectively, was calculated as reference time course. The time course of each voxel within the brain was extracted and then shifted from −3 TR to 3 TR (from −6s to + 6 s). The correlation coefficient between the shifted time course of each voxel and the reference time course was calculated at each TR, and then each voxel was assigned a value based on the time shift required for the maximal correlation coefficient to the reference time course. Finally, a time-shift map of each individual was acquired.

#### Dice coefficient (DC) analysis

Two radiologists (F.Z. and W.B.W., with 3 and 4 years of experience, respectively) independently traced the hypoperfusion regions (TTP) and time delay results in TSA for each patient. One traced the masks based on the TTP maps from DSC-PWI, and the other traced masks based on TSA delay results. Overlap was calculated using the DC, which calculates the ratio of the intersection with respect to the union of each pair of masks.

## Additional Information

**How to cite this article:** Ni, L. *et al*. The value of resting-state functional MRI in subacute ischemic stroke: comparison with dynamic susceptibility contrast-enhanced perfusion MRI. *Sci. Rep.* **7**, 41586; doi: 10.1038/srep41586 (2017).

**Publisher's note:** Springer Nature remains neutral with regard to jurisdictional claims in published maps and institutional affiliations.

## Figures and Tables

**Table 1:** Characteristics of cohort A (n = 31) of subacute stroke patients with BOLD and DSC-PWI data.

Patient No.	Age(y)/Gender/Time(d*)	Involved artery	Lesion on DWI	ΔTTP (s)	Volume (ml)			Overlap	
					TTP	TSA_(H)_	TSA_(G)_	TTP& TSA_(F)_	TTP &TSA_(G)_
A1^Δ^	53/F/5	L ICA-IS stenosis	L BG,CR	2.47	4701	6339	6280	0.5512	0.5361
A2^Δ^	72/F/4	R ACA stenosis; BA/PCA occlusion	R O, BG,CR	0.45	1663	1757	1820	0.3902	0.3743
A3^Δ^	39/M/8	L MCA stenosis	L BG,CR, Insular	2.00	3539	4474	4319	0.6814	0.6509
A4^Δ^	57/M/9	LMCA occlusion	L BG,CR	0.10	1880	2978	3001	0.3600	0.3545
A5^Δ^	61/M/6	L ICA-IS stenosis	L CR,F,P	1.83	5015	4601	4500	0.6934	0.6462
A6^Δ^	41/M/14	L MCA stenosis	L BG,CR,CO	2.43	5894	8702	8520	0.5267	0.5363
A7^Δ^	47/M/3	L MCA stenosis	L BG,CR	4.43	6618	5804	6000	0.7376	0.7509
A8^Δ^	25/M/12	R MCA stenosis	R BG	3.12	3620	3789	3691	0.6204	0.6732
A9^Δ^	58/M/6	L MCA stenosis	L BG,CR	1.53	4932	4608	4791	0.5023	0.5476
A10^Δ^	62/M/11	BA stenosis	Brainstem	0.60	1921	2123	2207	0.5396	0.5104
A11^Δ^	48/F/8	L MCA stenosis	L BG,CR	2.49	6002	6208	6165	0.7767	0.7825
A12^Δ^	48/M/6	R MCA stenosis	R Thalamus	3.10	6276	6498	6349	0.7194	0.7238
A13^Δ^	49/M/5	L MCA stenosis	L P,F	4.02	6546	6712	6697	0.5806	0.5902
A14^Δ^	46/M/7	L MCA stenosis	L BG,CR	3.58	4692	4980	5003	0.5783	0.5497
A15	54/M/4	MRA(−)	L BG	0.12					
A16	49/M/6	L MCA mild stenosis	L CR	0.18					
A17	68/M/13	L MCA mild stenosis	L BG	0.26					
A18	57/M/8	R MCA mild stenosis	R CR	0.15					
A19	63/M/3	MRA(-)	L CR	0.14					
A20	52/M/10	L ICA-IS stenosis	L BG	0.13					
A21	67/F/5	R ICA-IS mild stenosis	R BG,CR	0.31					
A22	80/M/12	L ICA-IS mild stenosis	L Thalamus	0.10					
A23	76/F/8	L MCA mild stenosis	L BG	0.17					
A24	55/M/3	MRA(-)	L BG	0.24					
A25	64/M/4	L MCA mild stenosis	L BG	0.52					
A26	58/M/4	L MCA mild stenosis	L CR	0.32					
A27	66/M/6	L MCA mild stenosis	L BG,CR	0.16					
A28	59/M/5	L ACA mild stenosis	Brainstem	0.12					
A29	54/F/12	L MCA mild stenosis	L BG,CR	0.31					
A30	60/M/8	L MCA mild stenosis	L BG,CR	0.29					
A31	51/M/6	L MCA mild stenosis	L BG,CR	0.11					

ICA: internal carotid artery; ACA: anterior cerebral artery; PCA: posterior cerebral artery; O: occipital lobe; F: frontal lobe; P: parietal lobe; MCA: middle cerebral artery; BA: basilar artery; VA:vertebral artery; IS:intracranial segment; BG: basal ganglia; CR: corona radiate; CO: centrum ovale; TTP: time to peak; TSA: time shift analysis. ^Δ^:patients showed TTP delay and time delay by TSA; d* means time to onset (day); _(H)_: referenced mean time course from healthy hemisphere (with normal DWI appearance) contralateral to the lesion; _(G)_: referenced mean time course from whole brain.

**Table 2:** Characteristics of cohort B (n = 9) of subacute stroke patients with only BOLD data.

Patient No.	Age(y)/Gender/Time(d*)	Involved artery	Lesion on DWI	Volume (ml)	
				TSA_(H)_	TSA_(G)_
B1^Δ^	67/M/5	L MCA stenosis	L CR,F,P	3723	3689
B2^Δ^	63/F/9	L ACA/VA/BA stenosis; R PCA occlusion	LBrachium Pontis	1347	1513
B3^Δ^	61/M/5	L MCA stenosis	L CR,F,P	4672	4546
B4^Δ^	42/M/14	LMCA occlusion	L CR	3687	3812
B5^Δ^	55/M/3	BA stenosis	L O	2019	2089
B6^Δ^	64/M/12	L MCA stenosis	L BG,CR,F	3387	3501
B7^Δ^	67/M/3	L MCA stenosis	L F,P,T	6218	6004
B8^Δ^	41/F/6	L MCA stenosis	L F,P,T	4376	4502
B9	53/M/4	LMCA stenosis	L BG	—	

ACA: anterior cerebral artery; PCA: posterior cerebral artery; O: occipital lobe; F: frontal lobe; P: parietal lobe; MCA: middle cerebral artery; BA: basilar artery; VA:vertebral artery; BG: basal ganglia; CR: corona radiate; TTP: time to peak; TSA: time shift analysis. ^Δ^:patients showed time delay by TSA; d* means time to onset (day); _(H)_: referenced mean time course from healthy hemisphere (with normal DWI appearance) contralateral to the lesion; _(G)_: referenced mean time course from whole brain.
